# Viral reprogramming of glial metabolism as a driver of neuroinflammation

**DOI:** 10.3389/fimmu.2025.1686774

**Published:** 2025-12-19

**Authors:** Tamara Rodrigues, Gabriel Salles Beltrão, Heloísa Girardi, Aguinaldo R. Pinto

**Affiliations:** Laboratory of Applied Immunology, Department of Microbiology, Immunology and Parasitology, Center for Biological Sciences, Federal University of Santa Catarina, Florianópolis, Brazil

**Keywords:** CNS, glial cells, inflammasome, metabolic reprogramming, neuroinflammation, neurotropic viruses

## Abstract

Considerable attention has been recently devoted to the involvement of immune cells in the central nervous system (CNS) during infections with neurotropic viruses, such as SARS-CoV-2, HIV-1, and ZIKV. These viruses are capable of infecting astrocytes and microglia, the main glial cells in the CNS, responsible for regulating neuronal activity. Here, we discuss how viral infections lead to metabolic reprogramming toward aerobic glycolysis in these cells, enhancing pro-inflammatory pathways, such as inflammasome activation, resulting in the secretion of inflammatory cytokines that favor the development of neuroinflammation. In this mini review, we discuss the pivotal interplay between metabolism and immunity towards viral pathogenesis in the CNS, pointing out the relevance of therapeutic strategies targeting both metabolic and immunological pathways to enhance antiviral and neuroprotective responses.

## Introduction

The role of immune cells in the central nervous system (CNS) in response to infection, injury or illness has recently been the focus of significant debate, as modulating the signaling and function of these cells has a high potential as a viable target for therapeutic interventions. Microglia are the resident macrophages of the CNS and serve as the first line of defense against foreign agents. Under normal physiological conditions, microglia perform essential support functions for the neural tissue. These functions include synaptic pruning during development and maintaining brain homeostasis through phagocytosis of parenchymal debris. They also continuously monitor the neural environment for signs of cellular damage or infections. When activated, microglia change their morphology to an ameboid state and secrete inflammatory cytokines (1–3). Astrocytes, the most abundant glial cells in the CNS, play a crucial role in maintaining neuronal health. In addition to their basic support functions, astrocytes are involved in a variety of essential processes. These include providing structural and metabolic support, regulating synaptic activity, controlling blood flow and water transport, and preserving the integrity of the blood-brain barrier (BBB). Furthermore, these cells secrete neurotrophic factors like the Brain Derived Neurotrophic Factor (BDNF) and the Glial cell line-Derived Neurotrophic Factor (GDNF), which are vital for the survival of dopaminergic neurons (3–5).

In recent years, it has become clear that metabolism and immune function are closely linked. Notably, each population of immune cells possess a particular metabolic program and pattern of nutrient utilization (6). Microglia and astrocytes can adapt their metabolic profile in response to changes in their microenvironment or disease conditions (5, 7, 8). Among pathological stimuli, viral infections induce significant metabolic reprogramming that is central to effective antiviral immune responses (9). These changes involve shifts in glucose, lipid, and amino acid metabolism, supporting energy-intensive functions such as cytokine production, clonal expansion, and viral clearance (10). Thus, the interplay between metabolism and immunity is critical in viral pathogenesis, underscoring the importance of therapeutic strategies that target both metabolic and immune pathways to enhance antiviral protection. This review explores how viral infections modulate the metabolism of glial cells, with a special focus on microglia and astrocytes, highlighting their metabolic adaptations that contribute to neuroinflammation.

## Glial immunometabolism in health and disease

Under physiological conditions, microglia and astrocytes utilize distinct yet complementary metabolic pathways to sustain their specialized roles. Through oxidative phosphorylation (OXPHOS), microglia maintain a metabolic profile that supports their homeostatic and immunoregulatory functions in the healthy brain (11). In this process, electrons derived from the oxidation of metabolic substrates are transferred through a series of protein complexes embedded in the inner mitochondrial membrane. The energy released drives the translocation of protons into the intermembrane space, creating an electrochemical gradient that is then utilized by adenosine triphosphate (ATP) synthase to phosphorylate adenosine diphosphate into ATP (12). This metabolic state is associated with low production of pro-inflammatory mediators and favors the expression of anti-inflammatory cytokines like interleukin-10 (IL-10) and transforming growth factor-beta (TGF-β), which contribute to immune tolerance within the CNS (13). Additionally, OXPHOS enables microglia to preserve redox homeostasis by fueling antioxidant systems, including glutathione synthesis (14). Thus, OXPHOS not only sustains the energetic needs of homeostatic microglia but also underpins their neuroprotective and immunoregulatory functions in the healthy brain.

**Figure 1:**
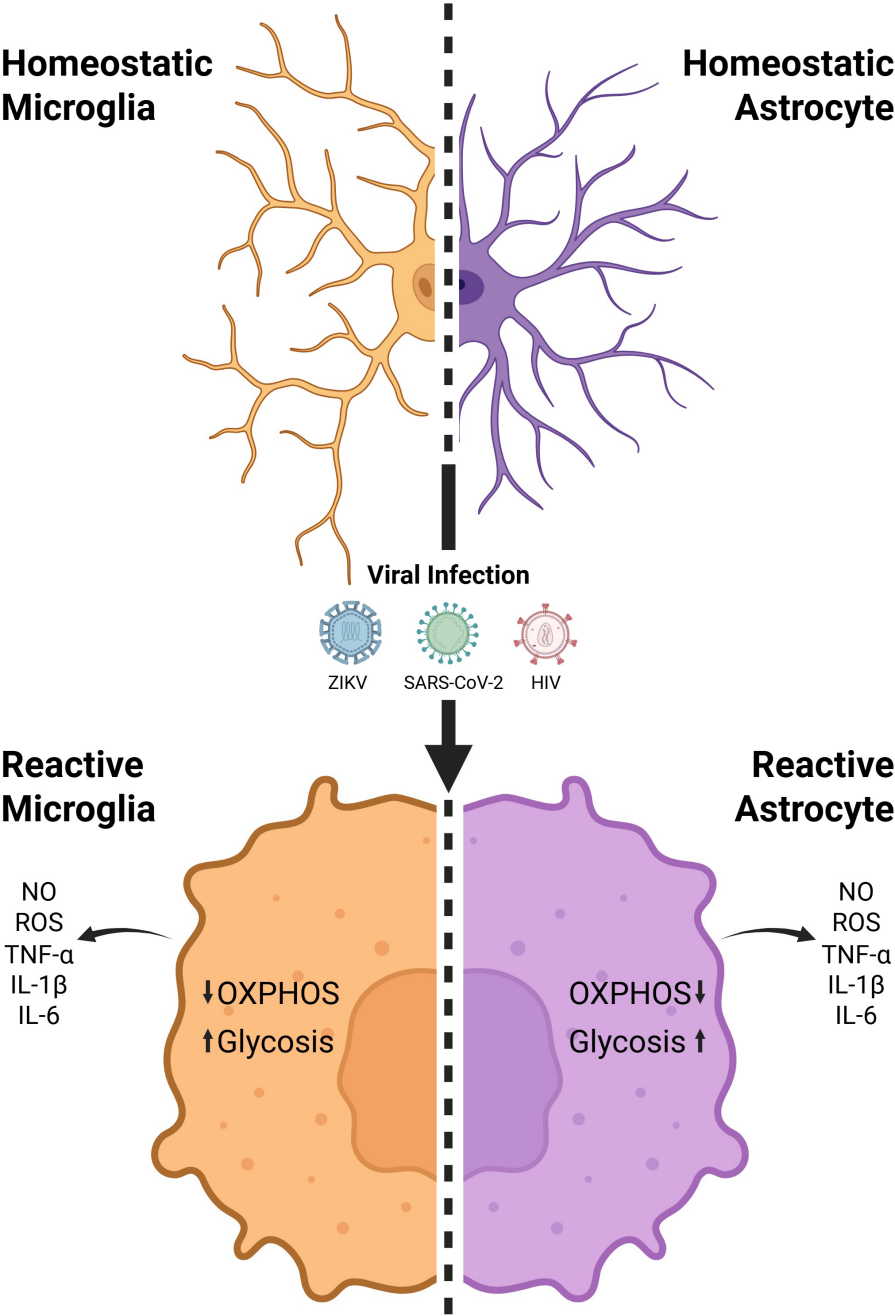
Viral infection-induced metabolic alterations in microglia and astrocytes drive neuroinflammation. Homeostatic microglia and astrocytes undergo metabolic and inflammatory reprogramming during viral infection with Zika virus (ZIKV), SARS-CoV-2, or Human Immunodeficiency Virus (HIV), characterized by decreased oxidative phosphorylation (OXPHOS), increased glycolysis, and production of nitric oxide (NO), reactive oxygen species (ROS), and pro-inflammatory cytokines (TNF-α, IL-1β, IL-6). Created in BioRender. Beltrao, G. S. (2025) https://BioRender.com.

In pathological conditions, microglia undergo metabolic reprogramming that shifts their primary energy production from OXPHOS to aerobic glycolysis driven by inflammatory stimuli (15) (**Figure 1**). This shift involves the inhibition of tricarboxylic acid (TCA) cycle enzymes, notably isocitrate dehydrogenase and succinate dehydrogenase. Blocking these enzymatic steps leads to the accumulation of citrate and succinate. Succinate acts as an immunometabolic signal by stabilizing hypoxia-inducible factor 1-alpha (HIF-1α), promoting the transcription of glycolytic enzymes such as hexokinase 2 (HK2), phosphofructokinase (PFK), and the glucose transporter GLUT1, as well as pro-inflammatory mediators including IL-1β, Tumor Necrosis Factor (TNF-α), and nitric oxide (NO). Additionally, inflammatory signaling pathways such as AKT-mTOR contribute to this response by enhancing the translation and stabilization of HIF-1α, thereby intensifying aerobic glycolytic flux and the sustained production of these inflammatory mediators (16, 17). Concurrently, mitochondrial fragmentation is exacerbated, and the expression of OXPHOS components is reduced, impairing cellular respiration. Although glycolysis is less efficient in generating ATP, it is activated for its rapid ATP production and its ability to supply biosynthetic intermediates essential for the synthesis of inflammatory mediators, including IL-1β, TNF-α, and NO. However, this reprogramming also results in elevated production of reactive oxygen species (ROS) and a decline in phagocytic capacity, consolidating a persistently pro-inflammatory, metabolically impaired phenotype that exacerbates oxidative stress, disrupts tissue homeostasis, and perpetuates CNS inflammation (18).

Astrocytes are metabolically specialized to support both neuronal activity and local immune responses. Under physiological conditions, they primarily rely on aerobic glycolysis, converting glucose into lactate, which is exported to neurons as an energy substrate through the astrocyte-neuron lactate shuttle (5). This metabolic strategy enables rapid ATP generation and the production of biosynthetic intermediates necessary for neurotransmitter recycling, ion homeostasis, and maintenance of synaptic function (19). In addition to glucose metabolism, astrocytes exhibit remarkable metabolic flexibility, engaging in fatty acid oxidation and glutaminolysis, particularly under conditions of increased energy demand or oxidative stress (20).

In pathological contexts, astrocytes undergo metabolic remodeling that transforms their functional profile. In chronic neuroinflammatory conditions, they shift into a reactive state marked by hypertrophy, altered transcriptional programs, and loss of homeostatic roles (21) (**Figure 1**). This transformation is often driven by microglial-derived proteins, including IL-1α, TNF-α, and complement component C1q, which collectively induce a reactive pro-inflammatory astrocyte (A1) (22). A1 astrocytes lose their neuroprotective capacities and begin secreting pro-inflammatory and neurotoxic molecules, such as complement C3, that exacerbate neuronal injury. Metabolically, these cells exhibit impaired OXPHOS and reduced fatty acid oxidation, accompanied by a compensatory increase in glycolytic flux and glutaminolysis. This shift compromises mitochondrial efficiency and antioxidant defenses, leading to elevated oxidative stress and impaired regulation of glutamate uptake, similar to the reactive pro-inflammatory microglia (23). As a result, neuronal excitotoxicity, synaptic dysfunction, and BBB disruption are intensified. Reactive astrocytes also contribute to glial scar formation, limiting tissue repair and facilitating chronic immune cell infiltration. Collectively, these changes are active contributors to disease progression, sustaining CNS inflammation and neurodegeneration (24).

Therefore, glial cells play an essential role in CNS homeostasis and are rapidly responsive to pathological stimuli, undergoing metabolic changes that lead to their activation. The microglial activation reflects their immune-sensing and inflammatory cytokine production, while astrocytic activation corresponds to their transition into reactive phenotypes (A1/A2) involving metabolic and functional reprogramming (25–27).

## Viral modulation of glial metabolism

Neurotropic viruses such as Zika virus (ZIKV), Severe Acute Respiratory Syndrome Virus 2 (SARS-CoV-2), and Human Immunodeficiency virus 1 (HIV-1) modulate the metabolism of the host cell to invade, persist, and replicate within the CNS (10, 28, 29). When infecting glial cells, such as microglia and astrocytes, neurotropic viruses modulate the intracellular metabolism of host cells, changing the energy production pathway from OXPHOS to glycolysis, decreasing the Pentose Phosphate Pathway (PPP), and increasing the TCA cycle (10, 28).

SARS-CoV-2 infects astrocytes via the Neuropilin 1 receptor (NRP1) and modulates the glycolysis pathway, reducing pyruvate and lactate levels, in addition to affecting the production of essential intermediates of the PPP. The glutaminolysis and TCA pathways also change in glial cells, leading to a decrease in glutamine and its metabolites, such as GABA, glutamate and alpha-ketoglutarate. These changes suggest a compromise in energy metabolism, mainly in the glycolysis and glutaminolysis pathways, which are crucial for neuronal support and cerebral homeostasis, as demonstrated in postmortem human brain slice cultures (30, 31). In the human microglial cell line HMC3, the SARS-CoV-2 S protein increases the oxygen consumption rate (OCR), indicating mitochondrial dysfunction (32). Such metabolic changes might be triggering the observed pro-inflammatory phenotype in these cells, characterized by the release of IL-1β, IL-6 and TNF-α (29).

The neuronal cells are the main target of ZIKV infection in the CNS. The excessive inflammatory response caused by this infection is responsible for neuroinflammation and neuronal damage, which can lead to complications such as congenital microcephaly, Guillain-Barré Syndrome, transverse myelitis, and meningoencephalitis (33). Infection of astrocytes by ZIKV activates MAPK and leads to the degradation of NMNAT2, and, therefore, a reduction in nicotinamide adenine dinucleotide (NAD+), favoring metabolic dysfunctions. Such a response is explained by nicotinamide adenine dinucleotide (NAD+) metabolism, which is highly dependent on the degradation of MAPK-mediated nicotinamide adenylyltransferase 2 (NMNAT2) (34). The described metabolic dysfunctions trigger axon degeneration, indicating a critical impact on the pathogenesis of ZIKV-induced microcephaly. In addition, they interrupt the glycolytic flow in the TCA cycle in human primary astrocyte and microglia *in vitro* models, leading to mitochondrial dysfunction, which triggers inflammation, with the release of pro-inflammatory cytokines, such as IL-6, IL-1α, IL-4, IL-10, IL-8, and TNF-α, and resulting in neuronal cell death (35–37).

HIV is strongly associated with neurological complications and cognitive impairment. The HIV Tat protein accelerates cell damage in the CNS, in addition to activating astrocytes and damaging surrounding neurons in transgenic mouse models (38). Astrocytes’ metabolic response to the Tat protein is the alteration of aerobic glycolysis for mitochondrial respiration, mediated by the mitochondrial Ca2+ uniporter (MCU), which regulates the uptake of Ca2+; however, the direction of the MCU rescues glycolysis and normalizes the levels of extracellular lactate in the astrocyte. The Tat protein is also involved in the energy metabolism of glial cells, decreasing the activity of lactate dehydrogenase in activated astrocytes, which has led to a reduction in extracellular lactate levels (39, 40). HIV carriers have an imbalance in the levels of glutamate, a more abundant excitatory neurotransmitter, and GABA, an inhibitory neurotransmitter in the CNS, which can generate complications in brain functions, including alterations in synaptic signaling, cognition, motor stimuli and pain (28). Although robust data on metabolic reprogramming in glia are available mainly for ZIKV, HIV-1 and SARS-CoV-2, we have compiled a comprehensive summary in **Table 1**, including other neurotropic viruses (HSV-1, EBV, CMV, RABV), in order to also highlight knowledge gaps. For this reason, we will focus our discussion on ZIKV, HIV-1, and SARS-CoV-2, the viruses with the most consistent and well-documented evidence of glial metabolic reprogramming.

**Table 1:** Metabolic signatures and neurological manifestations induced by neurotropic viruses.

Neurotropic viruses	Permissive CNS cell types	CNS manifestation	Metabolic signatures	Inflammasome activation profile	References
SARS-CoV-2	Astrocyte Microglia	Headache Ischemic stroke Seizures Delirium Anosmia Ageusia Encephalopathy Paralysis	↓OXPHOS ↑Glycolysis ↓PPP ↓TCA ↓Glutaminolysis	Microglia: NLRP3-dependent inflammasome activation Astrocytes: NLRP2-dependent inflammasome activation	(10, 28–30, 32, 41–43)
ZIKV	Astrocyte Neuron	Congenital microcephaly Guillain-Barré Syndrome Transverse myelitis Meningoencephalitis	↑MAPK ↓TCA	Microglia: NLRP3 dependent inflammasome activation Astrocytes: Non-canonical caspase-1 activation with no evidence of classical inflammasome platform.	(10, 28, 32, 33, 35–37, 44–46)
HIV-1	Astrocyte Microglia	Mild cognitive Motor disorder	↓Glycolysis ↑OXPHOS	Microglia: NLRP3 dependent inflammasome activation Astrocytes: NLRP3-dependent inflammasome activation	(10, 28, 38–40, 47, 48)
HSV-1	Astrocyte Microglia	Cognitive dysfunction Personality changes Aphasia Seizures	↑Aerobic glycolysis ↑ROS ↑Oxidative Damage	Microglia: NLRP3 dependent inflammasome activation Astrocyte: Positive expression of NEK7, essential mediator of NLRP3 activation	(49–54)
EBV	Astrocyte Microglia Oligodendrocytes	Parenchymal damage Intracranial hypertension Fever Headache Vomiting Signs of meningeal irritation	↑Glycolysis	No glial inflammasome evidence available	(55–57)
CMV	Astrocyte Microglia	Sensorineural hearing loss Seizures Epilepsy	↑Aerobic oxidation ↓Glycolysis	No glial inflammasome evidence available	(57–59)
RABV	Astrocyte Microglia Neuron	Agitation Hypersalivation Hydrophobia	↑Oxidative stress ↑ROS ↑NO	No glial inflammasome evidence available	(52, 60, 61)

SARS-CoV-2, Severe Acute Respiratory Syndrome Coronavirus 2; ZIKV, Zika Virus; HIV-1, Human Immunodeficiency Virus type 1; HSV-1, Herpes Simplex Virus type 1; EBV, Epstein–Barr Virus; CMV, Human Cytomegalovirus; RABV, Rabies Virus.

## Inflammasomes at the interface between metabolism and viral neuroinflammation

Inflammasomes are cytosolic multiprotein complexes that play a crucial role in orchestrating inflammatory responses in multiple organs, including the CNS (62–64). Within the CNS, inflammasomes are expressed by a subset of glial cells, such as microglia and astrocytes, and, interestingly, have also been detected in neurons (65, 66).

Among the inflammasome complexes, NLRP3 is the most abundant and extensively investigated in the CNS, given its relevance to neuroinflammation and neurological disorders (64). The activation of NLRP3 recruits the adaptor molecule ASC and triggers the release of the pro-inflammatory cytokines IL-1β and IL-18, processed by caspase (casp-1), which compromises brain homeostasis and is associated with cognitive deficits and depressive-like behavior (64, 67, 68). Inflammasome activation can also induce pyroptosis, an inflammatory cell death mediated by gasdermin D (GSDMD), a pore-forming molecule, further aggravating neuronal damage (65, 69–71).

The activation of inflammasomes in glial cells remains controversial. In microglial BV2 cells, intrinsic NLRP3 activation can occur in response to diverse stimuli (72, 73). Astrocytes, in contrast, are traditionally viewed as being activated indirectly by microglia, which sense danger signals, activate NLRP3, and release IL-1β and IL-18, thereby promoting the A1 reactive astrocyte phenotype (27, 74). However, more recent evidence shows that astrocytes can also intrinsically activate inflammasomes. In Alzheimer’s disease, microglia activate pyroptosis via the canonical NLRP3 pathway, while astrocytes utilize alternative caspase-8 or caspase-4 dependent pathways, a process associated with neuronal loss (65). In rodent models of prion disease, GFAP+ astrocytes express NLRP3 and exhibit GSDMD cleavage, contributing to inflammation and neuronal death (75).

Other studies show that AIM2 inflammasome could also be activated in astrocytes. In the experimental autoimmune encephalomyelitis model, inflammasome activation occurs mainly in resident astrocytes, depends on AIM2, and does not involve cell death and a robust IL-1β secretion, indicating a less inflammatory function (76). Interestingly, evidence also points to homeostatic roles for inflammasomes in the CNS. Hippocampal ASC clusters disassemble during cognitive tasks, and the deletion of astrocytes casp-1 alters neuronal excitability and memory, supporting a regulatory role in brain physiology (77).

Oxidative stress plays a central role in NLRP3 inflammasome activation, with ROS acting as a major upstream signal, including in neuronal cells (78–83). Metabolic shifts, such as increased glycolysis, accumulation of succinate, and compromised mitochondrial function, promote NLRP3 inflammasome activation, positioning it as a central mediator between bioenergetic stress and inflammation (84–86). For instance, the M2 isoform of muscle pyruvate kinase (PKM2), which catalyzes the final reaction of the glycolytic pathway, induces NLRP3 and AIM2 inflammasome activation (87). Furthermore, succinate accumulation acts as an inflammatory signal, inducing IL-1β production through HIF-1α (86) and ROS generation (88). In addition to these upstream effects, there is growing evidence that inflammasome activation also remodels glial metabolism, establishing a bidirectional axis between metabolism and inflammation. In microglia, NLRP3 activation reinforces a pro-inflammatory bioenergetic state, reducing mitochondrial flexibility, while its inhibition increases OXPHOS and reprograms glial metabolism (89). The NLRP3 inflammasome can induce glycolysis via 6-phosphofructo-2-kinase/fructose-2,6-bisphosphatase 3 (PFKFB3), leading to microglial polarization towards the M1 phenotype (85, 90). In addition, casp-1 cleavage in astrocytes regulates metabolic pathways by modulating pyruvate flow into mitochondria, directly influencing glycolysis, TCA, and mTORC activity (46).

Viral infection in the CNS is a potent inducer of inflammasome-mediated neuroinflammation. For example, ZIKV can infect and replicate in glial cells, including microglia, inducing NLRP3 activation, resulting in the release of mature IL-1β (44, 67). This inflammatory response has deleterious consequences for neurodevelopment, as ZIKV infection can lead to severe birth defects, such as microcephaly, by affecting neural progenitors and impairing brain development (67, 91). In addition, the P2X7 receptor, a key upstream activator of the NLRP3 inflammasome, is highly expressed in the brains of infected mice, contributing to neuronal loss, neuroinflammation, and brain anomalies by inhibiting the neuroprotective AKT/mTOR pathway (67). However, a recent study showed that ZIKV activates casp-1 in primary astrocytes even in the absence of other inflammasome signals, and astrocytes deficient for casp-1 and caspase-11 (*Casp1/11-/-)* become more permissive to ZIKV replication due to glycolytic hyperactivation, indicating that inflammasomes also participate in antiviral control in these cells (46). Therefore, although inflammasomes can trigger potentially deleterious inflammatory responses, their components also perform non-canonical functions, such as modulating cellular metabolism and controlling viral replication.

Regarding SARS-CoV-2, the S1 protein can cross the BBB (80) and induce neuroinflammation, even in the absence of productive infection, with S1 binding to microglial TLR4 receptors (81, 92). It is well established that SARS-CoV-2 activates the NLRP3 inflammasome and contributes to disease severity (93–95). SARS-CoV-2 infection in transgenic animal models results in microglial activation and upregulation of the NLRP3 inflammasome in the brain, accompanied by an increase in casp-1 activity and IL-1β production (96, 97). Similarly, NLRP3 was active in the brains of COVID-19 cases, associated with microglial dysfunction, significant morphological changes, and microglial degeneration (41, 42, 98). Metabolic failure and mitochondrial damage, associated with the efflux of Cytochrome c (Cytc), are also observed in areas of the brain affected by vascular inflammation related to viral antigens (42). The consequences of NLRP3 activation include cognitive impairment, neuronal loss, and neuroinflammation (96). This inflammatory cycle can become chronic, leading to persistent microglial dysfunction and neurodegeneration, as observed in SARS-CoV-2 and HIV-1 infections (99–103). Thus, inflammasomes can act as sensors of cellular metabolism and also modulate it: when acutely activated, they can contribute to the control of viral infections; however, their persistent activation can promote neuroinflammation, lasting neural damage, and compromise brain function.

## Therapeutic implications and future directions

As glial cells alter their metabolic programs during inflammation, especially changing from OXPHOS to glycolysis (104), these metabolic changes offer actionable targets for therapeutic intervention in CNS infections. To modulate the OXPHOS pathway, therapeutic targets have focused on promoting an anti-inflammatory phenotype of microglia and restoring mitochondrial function, aiming to enhance the use of the OXPHOS pathway and consequently contribute to neuroprotection and brain recovery. On the other hand, the main therapeutic targets of glycolysis focus on inhibiting specific enzymes, such as PFKFB3 and PKM2, thereby decreasing microglial pro-inflammatory activation by restraining hyperactive glycolysis (105–107). The modulation of these two pathways has been presented as a potential therapeutic strategy in neurodegenerative diseases, such as Alzheimer’s Disease (AD) and Multiple Sclerosis (MS) (108).

Pharmacological agents have been used as metabolic interventions to limit viral replication and preserve neuronal function. Glycolysis inhibitors, such as 2-DG, a drug that structurally mimics glucose, are promising against excessive inflammation, in addition to metabolic modulators that transform immune metabolism towards OXPHOS, such as metformin and AMPK activators. Additionally, ketogenic diets have shown promising responses in neuroprotection in viral infections in the CNS (10, 109). A clinical trial using 2-DG along with standard medications for concomitant treatment of moderate to severe COVID-19 resulted in faster recovery of patients as measured by oxygen levels, time to discharge and normalization of vital signs, as well as by increment on tolerance to treatment as revealed by the presence of moderate side effects (110). However, CNS viral disease pathogenesis requires therapeutic strategies that integrate glial cell activation and metabolic control. The decrease or remission of neuroinflammation caused by viral invasion can help relieve symptoms and reduce severe neurological sequelae. The currently available antiviral drugs are not effective for CNS infections, demanding a deeper understanding of viral neuropathogenesis, improvement of strategies for the treatment and diagnosis of these infections (10, 28).

In neurodegenerative diseases, modulation of microglial metabolism has become a possible therapeutic strategy to restore cellular homeostasis and reduce neuroinflammation. For instance, metformin and rapamycin are pharmacological agents that inhibit the AKT-mTOR-HIF-1α and AMPK-mTOR-HIF-1α pathways, contributing to a decrease in energy production by glycolysis while increasing production by the OXPHOS pathway, consequently reducing the production of pro-inflammatory cytokines. Metformin acts on the mTOR pathway by targeting AMPK, while rapamycin directly inhibits mTOR activity by binding to mTORC1 (17, 109). The Triggering Receptor Expressed in Myeloid Cells 2 (TREM2) could be another target to modulate the mTOR pathway in microglia, in the absence of which, it may result in defective glycolysis. However, there are still not enough studies on pharmacological agents that modulate TREM2 directly, making it difficult to understand the functionality of TREM2 and its precise association with neurological disorders (17, 111). In addition, modulating glucose microglial metabolism remains incompletely understood regarding its efficacy in changing microglial phenotype, immunological tolerance and the neuropathogenesis of neurodegenerative diseases (17). Therefore, current data highlight the need for further studies aimed at therapeutic development in neuroinflammation caused by viral infections.

## Final remarks

This review points out that viral infections caused by neurotropic viruses, such as HIV, SARS-CoV-2, and ZIKV, modulate the metabolism of glial cells, especially microglia and astrocytes, causing metabolic adaptations that contribute to neuroinflammation through inflammatory mechanisms, such as the activation of inflammasomes. Under physiological conditions, microglia and astrocytes utilize distinct metabolic pathways, with the former utilizing OXPHOS while astrocytes are predominantly glycolytic, however, under pathological conditions such as viral infections, microglia shift their primary energy production to aerobic glycolysis and adopt a more glycolytic metabolism, similar to astrocytes. Aerobic glycolysis favors the activation of inflammasomes, which are often associated with the pathogenesis of these diseases, inducing an exacerbated inflammatory response. This inflammatory cycle can lead to the dysfunction of glial cells, inducing neurodegeneration in viral infections.

Despite substantial advances, important limitations remain in the study of glial cells. Differences between human and mouse glia, the restricted access to primary human CNS tissue, and the heterogeneity of glial states across brain regions limit the generalizability of many mechanistic findings (112–114). In addition, although *in vitro* models using isolated glial populations are essential for dissecting cell-intrinsic pathways with experimental precision, they only partially capture the dynamic interactions that occur between microglia and astrocytes *in vivo*. Microglia and astrocytes are known to communicate through various molecular signals, such as the release of metabolites and cytokines, and their reciprocal signaling significantly influences neuroinflammation (115, 116). For instance, the metabolic state and cytokine profile of microglia can determine whether astrocytes adopt protective or neurotoxic reactive phenotypes (27). Thus, complementary approaches, including iPSC-derived glia, co-cultures, and 3D organoids, are therefore valuable to contextualize how inflammasome signaling and metabolic reprogramming emerge in more physiologically integrated environments.

Although there is a limitation in selectively modulating the metabolism of specific cells, the similar metabolic changes in astrocytes and microglia lead us to believe that targeting cellular metabolism may be a promising neuroprotective therapeutic strategy and may be important for the development of biomarkers. Further mechanistic and translational research is warranted to clarify these functions and explore these metabolic pathways as therapeutic targets in neuroinflammation.
